# The Sexual Question

**Published:** 1908-10

**Authors:** 


### BOOKS AND AUTHORS.

**The Sexual Question.** A Scientific, Psychological, Hygienic and Sociological Study for the cultured classes. By **August Forel,** M.D., Ph.D., LL.D., formerly Professor of Psychiatry at and Director of the Insane Asylum in Zurich, Switzerland. English adaption by **C. F. Marshall,** M.D., F.R.C.S., late Assistant Surgeon to the Hospital for Diseases of the Skin, London. Octavo, pp. 536. Illustrated. New York: Rebman Company.

Professor Forel in this work presents a careful philosophical treatise of the sexual question. He considers the subject in its relation to suggestion, money and property, to the external conditions of life, to law, medicine, morality, politics, political economy, pedagogy and art. As a result of his study he arrives at some utopian ideas on the ideal marriage of the future.

First, the youth of both sexes will have been thoroughly educated as to the meaning of the sexual appetite. They will speak openly on sexual subjects to their masters and parents and to their companions of the opposite sex. The ideal marriage will reduce useless luxury and conventional formality to a minimum. Servants will not have the same position in the families as at present. He believes in matriarchism. This he defines is the legal privilege of the management of the family conferred on the wife, who is in reality the center of the family.

There is much in this work to commend. But there is much with which many will disagree. For instance: of this country we find the following, "since the emancipation of negroes has caused domestic servants in the United States to become expensive luxuries, family life has to a great extent been replaced by life in hotels and boarding houses, and this has furnished another reason for avoiding conception and large families. The European race of North America will diminish and become gradually extinguished and will soon be replaced by Chinese or negros."

After reading this on page 332, we must confess that our respect for the prognostics of the author diminished. For his facts and their presentation we have only praise.

